# *Potato leafroll virus* reduces *Buchnera aphidocola* titer and alters vector transcriptome responses

**DOI:** 10.1038/s41598-021-02673-6

**Published:** 2021-12-14

**Authors:** MacKenzie F. Patton, Allison K. Hansen, Clare L. Casteel

**Affiliations:** 1 Department of Plant Pathology, University of California, Davis, CA 95616 USA; 2 Plant Pathology and Plant-Microbe Biology Section, School of Integrated Plant Science, Cornell University, Ithaca, NY 14850 USA; 3 Department of Entomology, University of California, Riverside, CA 92521 USA

**Keywords:** Ecology, Molecular ecology

## Abstract

Viruses in the *Luteoviridae* family, such as *Potato leafroll virus* (PLRV), are transmitted by aphids in a circulative and nonpropagative mode. This means the virions enter the aphid body through the gut when they feed from infected plants and then the virions circulate through the hemolymph to enter the salivary glands before being released into the saliva. Although these viruses do not replicate in their insect vectors, previous studies have demonstrated viruliferous aphid behavior is altered and the obligate symbiont of aphids, *Buchnera aphidocola,* may be involved in transmission. Here we provide the transcriptome of green peach aphids (*Myzus persicae*) carrying PLRV and virus-free control aphids using Illumina sequencing. Over 150 million paired-end reads were obtained through Illumina sequencing, with an average of 19 million reads per library. The comparative analysis identified 134 differentially expressed genes (DEGs) between the *M. persicae* transcriptomes, including 64 and 70 genes that were up- and down-regulated in aphids carrying PLRV, respectively. Using functional classification in the GO databases, 80 of the DEGs were assigned to 391 functional subcategories at category level 2. The most highly up-regulated genes in aphids carrying PLRV were cytochrome p450s, genes related to cuticle production, and genes related to development, while genes related to heat shock proteins, histones, and histone modification were the most down-regulated. PLRV aphids had reduced *Buchnera* titer and lower abundance of several *Buchnera* transcripts related to stress responses and metabolism. These results suggest carrying PLRV may reduce both aphid and *Buchnera* genes in response to stress. This work provides valuable basis for further investigation into the complicated mechanisms of circulative and nonpropagative transmission.

## Introduction

Aphids are a member of the superfamily Aphidoidea, are distributed world-wide, and cause major damage to global agricultural^1^. Despite there being over 4000 species, only about 400 are known as significant pests^2^. Aphids are effective pests partially because they do not require sexual reproduction and can use parthenogenesis to quickly increase their numbers^1,2^. Another aspect of aphid biology that makes them an effective pest is their host range. Although many aphids are very specialized herbivores, only feeding on a few related species, some species feed on many taxa of plants. *Myzus persicae* is one of these polyphagous pests, feeding on over 40 different families, including Solanaceae^1,2^.

Along with causing direct feeding damage, aphids are important plant virus vectors, representing over 50% of all known insect vectors for plant viruses^3–5^. Increasing evidence has shown that plant viruses alter vector host finding, dispersal, and inoculation through changes in host physiology, however the underlying mechanisms are largely unknown^6–8^. Recent evidence suggests that viruses may also directly affect aphid biology^9–12^. For example, *Rhopalosiphum padi* that had been fed an artificial diet with purified *Barley yellow dwarf virus* (BYDV) virions prefer non-infected host plants, while virus-free *R. padi* prefer infected hosts^9^. By separating the virus from the host plant the authors demonstrate that the virus alone can impact insect behavior.

Although their life history or host may change, all aphids depend on *Buchnera aphidicola* as their primary obligate endosymbiont^13^. *Buchnera* provide the aphid with essential amino acids and nutrients that are limited in the aphid’s diet^14–17^, and because of this aphids can no longer survive without *Buchnera.* For example, when *Buchnera* is reduced by using antibiotics, studies have shown lower body mass, lower fecundity, and changes to feeding behavior^18,19^. As *Buchnera* co-diversified with aphids over time^13,20^, essential components of the *Buchnera* genome have also been lost^21,22^. Because of this *Buchnera* also depends on aphids for survival, living inside special aphid cells, known as “bacteriocytes”. Previous studies have speculated *Buchnera* may have a role in aphid transmission of plant viruses^23–27^. Specifically, the *Buchnera* chaperone protein GroEL, a homologue from *Escherichia coli*^28^, has been implicated in transmission for a number of viruses^23,24,27,29–32^. Direct interactions are thought to be unlikely due to the spatial separation of bacteriocytes and circulating virions^23^, however, GroEL from *Buchnera* is found in aphid saliva and has been shown to trigger plant defenses and reduce aphid fecundity using transgenic plants^33,34^.

*Potato leafroll virus* (PLRV) is a positive sense ssRNA virus and the type member of the genus *Polerovirus* (family *Luteroviridae*). PLRV is phloem limited and transmitted in a circulative nonpropagative manner. This means the virus particles will travel across the gut membrane on specific receptors into the insect hemolymph. From here it will traverse to the salivary gland and duct so that it may be injected back into the phloem tissue^6,35^. Previous studies have shown that aphid vectors prefer to settle on plants infected with PLRV and that insect vectors have higher fecundity when feeding on these plants compared to controls^36,37^. Recently we demonstrated PLRV induces changes in plant nutrients and defenses in infected host plant^38^, however, the impacts of these changes on symbiont-aphid interactions are unknown. To address this lack of knowledge we examined changes in the transcriptome of *M. persicae* with and without PLRV, *Buchnera* titer, and changes in aphid and *Buchnera* transcripts from aphids feeding on PLRV-infected plants. By providing evidence that nonpropagative circulative plant viruses can affect insect vectors through changes in the transcriptome and alter *Buchnera* titer, our study will contribute to growing knowledge of the insect microbiome at a plant–insect interface.

## Methods

### Plant and insect growth conditions

*Solanum tuberosum* were propagated using leaf-bud cutting from cv. Désirée^39^ in laboratory experiments. Plants were grown in growth chambers under controlled conditions (25/23 °C day/night with a photoperiod of 16/8 h day/night). Non-viruliferous and viruliferous aphid clones of a potato-adapted red strain of *Myzus persicae* were reared under controlled conditions (25/23 °C day/night with a photoperiod of 16/8 h day/night) on healthy potato. We confirmed our colony was free of secondary symbionts using universal primers for the bacteria 16S–23S ribosomal RNA intergenic spacer region (Supplementary Table S1). All experiments were conducted in the same environmental chambers and conditions, so there were no environmental differences in treatments (25/23 °C day/night with a photoperiod of 16/8 h day/night).

### Pathogen infection

*Agrobacterium tumefaciens* (LBA4404) containing the infectious clone of PLRV^40^ was grown at 28 °C in LB broth (+ 10 mM MgSO_4_), with kanamycin (50 µg/mL), carbenicillin (100 µg/mL) and rifampicin (50 µg/mL) for selection. After 24 h, bacteria were centrifuged to concentrate and resuspended in 10 mM MgCl_2_. One-week-old *S. tuberosum* were inoculated at an optical density (OD) of 0.70. Three weeks post infection, tissue was collected from all plants, RNA was extracted using the SV Total Isolation Kit as per manufacturer’s instructions (Promega, Madison, WI, USA), and cDNA was synthesized using 1500 ng of total RNA and random hexamers (20 ng/µL) with the SMART^®^ MMLV as per manufacturer’s instructions (Takara Bio USA, Mountain view, CA, USA). cDNAs were used in PCRs with PLRV specific primers (F-5’ATGAGTACGGTCGTGGTT-3’ and R-5 ‘CTATTTGGGGTTTTGCAAAGC-3’). A set of uninfected potato cuttings were grown at the same time as the plants above to serve as controls. After systemic plant infection was verified plants were immediately used in experiments.

### RNAseq, qRT-PCR, and qPCR aphid experiments

One week after infection was verified, five adult aphids were placed on the first fully expanded leaflet of three infected and three healthy plants. After 24 h, all adults were removed and 20 larvae were left to develop. Seven days later all aphids were at the same developmental stage. Ten adult aphids were collected into a tube from each plant (N = 3 plants with 10 aphids per plant) and immediately frozen in liquid nitrogen until use in RNAseq experiments. The entire experiment was repeated a second time for confirmation of RNAseq results using qRT-PCR and to examine the titer of the bacterial symbiont, *Buchnera*, in aphids. For this experiment 5 aphids were collected for RNA extraction and 5 aphids were collected for DNA extractions from each plant. We also prepared 6–7 plants for each treatment instead of 3 (N = 6–7 with 5 aphids per plant), however all other methods were the same.

### RNA and DNA isolation from aphids

RNA was extracted from aphid tissue collected in the first two experiments as described above. The RNA concentration and purity were measured using a NanoDrop. The integrity of RNA was confirmed using the Bioanalyzer 2100 system (Agilent, Santa Clara, CA, USA). DNA was extracted from aphid tissue collected in the second experiment using cetyl trimethylammonium bromide. The integrity of DNA was confirmed using an agarose gel. The DNA concentration and purity were measured using a NanoDrop (Thermo Fisher Scientific, Waltham, MA, USA).

### Library preparation, and sequencing

Sequencing libraries were prepared using a multiplexing library protocol^41^. Briefly, oligo(dT) 25 Dynabeads were used to purify mRNA, which was then fragmented, and the first-strand cDNA was synthesized using random primers, dNTP, and reverse transcriptase. The second-strand was synthesized using a dUTP mix, DNA Polymerase I, and RNase H, ends repaired, and adenylated. The cDNA fragments were ligated to adapters, selectively enriched by PCR, and purified using the AMPure XP beads. The library quality was assessed using the Agilent Bioanalyzer 2100 system and sequenced using an Illumina HiSeq 2000 instrument.

### Read mapping, differential expressed gene (DEG) analysis, and Gene Ontology (GO) classification

RNA-Seq data were analyzed using RStudio (Version 1.1.383) and Bioconductor according to Anders et al. (2013) with some modifications^39^ (See Supplementary Fig. S1). Sequence quality was determined, trimmed, and poor-quality reads removed using ShortRead^42,43^ and FastQC^44^. Reads were mapped to the *Myzus persicae* clone G006 genome v2.0 from AphidBase^45^ using TopHat2^46^. Mapped reads were assigned to genes and counted with HTSeq^47^, and normalized by size factors obtained from the negative binomial-based DESeq2 package^48^. Gene annotation files were downloaded from NCBI. After normalization, clusterization profiles of the samples were assessed by hierarchical clustering (with Euclidean distance metric and Ward´s clustering method) and principal component analysis (PCA). Differentially expressed genes (DEGs) between infected and control treatments were identified using DESeq2^48^. Genes with False Discovery Rate (FDR)-corrected p-values ≤ 0.1 were classified as differentially expressed. For Gene ontology (GO) analysis, Blast2GO software^49^ was utilized for annotation as previously described^50^.

### qRT-PCR

cDNA was synthesized using random hexamer (20 ng/µL) and quantitative RT-PCR (qRT-PCR) was performed. Transcript abundance was quantified for the *M. persicae* genes, *Hsp68-like* (MYZPE13164_G006_v1.0_000070430.1) and *M. persicae* cuticle protein5-like (*Mpcp5-like)* (MYZPE13164_G006_v1.0_000133030.2), and for the *Buchnera* genes, *argE (BUMPUSDA_CDS00542), dnaK (BUMPUSDA_CDS00441),* and *groEL (BUMPUSDA_CDS00567),* using gene specific primers (Supplementary Table S1). qRT-PCR reactions were carried out using SYBR green PCR master mix (Applied Biosystems, Carlsbad, CA, USA), in an CFX384 instrument (Bio-Rad, Hercules, CA, USA). Three technical replicates were performed for each individual sample, and a digital pipette was used for all pipetting. Relative transcript abundance was calculated utilizing a standard curve produced from tenfold series dilution of cDNA synthesized from 1000 ng/μL of total RNA according to the standard curve method (Applied Biosystems, Carlsbad, CA, USA). Technical replicates of raw CT values were averaged and transcripts of interest were normalized to the house-keeping transcript ribosomal protein L7 *rpl7* for aphids or the 50S ribosomal subunit gene *rpIN* for *Buchnera*, as previously described^51–53^.

### *Buchnera* titer

*Buchnera* titer here is defined as the ratio of *Buchnera* single copy genes to aphid single copy genes. To determine *Buchnera* titer in whole aphid bodies we used qPCR and measured the ratio of a single copy *Buchnera* gene (*rplN*) to a single copy aphid gene (*RPL7*). qPCR reactions were carried out using SYBR green PCR master mix (Applied Biosystems, Carlsbad, CA, USA), in the CFX384 instrument (Bio-Rad, Hercules, CA, USA). Reactions were performed in triplicate for each sample, and the average was used for analysis. Relative abundance was calculated utilizing a standard curve produced from tenfold serial dilution of DNA.

### Statistical analyses

RNAseq data analyses were performed as described above. All statistical analyses for qRT-PCR and qPCR were determined using a *P* < 0.05. Analysis of variance (ANOVA) was used to determine significant difference in transcript abundance. For *Buchnera* titer single factor ANOVA was used to determine difference in relative abundance. The statistical analyses were performed using JMP 8 software (SAS Institute, Cary, NC, USA).

### Compliance statement

All research in this paper complied with relevant institutional, national, and international guidelines and legislation.

## Results

### Differential gene expression in the presence of PLRV

**Figure 1:**
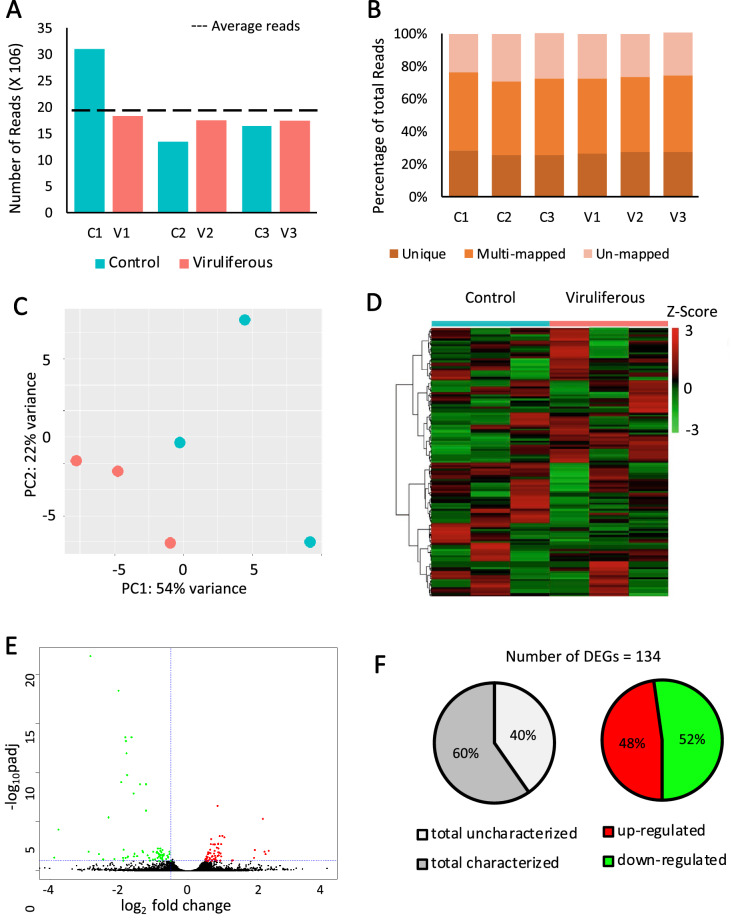
Overview of *Myzus persicae* transcriptome after *Potato leafroll virus* (PLRV) acquisition. **(A)** Number of paired-end reads generated for each library by Illumina HiSeq sequencing. The dashed line represents the average of paired-end reads from all 6 libraries. **(B)** Proportion of uniquely mapped, multimapped, and unmapped reads obtained for each library. Reads were mapped in the *Myzus persicae* clone G006 genome (AphidBase). **(C)** Principal component analysis of normalized count data from all samples. (D) Hierarchical clustering analysis of normalized count data z-scores exhibited by differentially expressed genes (DEGs) within each sample. **(E)** Volcano-plots of − log10p and log2FC exhibited by each gene in viruliferous aphids compared to controls. Up- and down-regulated genes are presented in red and green, respectively. **(F)** Numbers of up- and down-regulated DEGs in viruliferous aphids in comparison to control aphids. DEGs were identified using DESeq2 and defined by |log2FC|≥ 0.5 and false discovery rate (FDR)-corrected p-value ≤ 0.1. C = control aphids without virus; V = viruliferous aphids carrying PLRV, *p* = FDR-corrected p-value.

Over 150 million paired-end reads were obtained through Illumina sequencing, with an average of 125,815, 247 100-bp reads per library (Table 1, Fig. 1A). A higher number of average reads were sequenced in control 1 compared to other samples. Nevertheless, reads mapped to the target genome in the same relative proportions across treatments (Fig. 1B) revealing that the sample was not contaminated with reads other than for the target genome. Further, read counts were normalized before differential gene analyses (see methods) accounting for variation in library sizes among treatments. About 73% of the reads mapped to the *M. persicae* reference genome, with around only 27% being uniquely mapped (Table 1, Fig. 1B). In order to examine biological variability, a principal component analysis (PCA) of the normalized count data was performed (Fig. 1C). The first component of variance separated samples by treatments and accounted for 54% of the variance. Hierarchical clustering confirmed PCA results in visual representation of DEG expression (Fig. 1D). The transcriptome of viruliferous *M. persicae* was compared to the transcriptome of virus-free *M. persicae* using the negative binomial-based DESeq2^48^. Overall, 96 differentially expressed genes (DEGs) were detected using an FDR adjusted p-value ≤ 0.05 and log2 fold change (FC) ≥ 0.5, however, by relaxing our FDR adjusted p-value to ≤ 0.1 we were able to include 38 additional DEGs (134 DEGs total included; Fig. 1E; Supplementary Table S2). The presence of PLRV in the aphid vector caused a down-regulation of 70 genes and an up-regulation of 64 genes (Fig. 1F; FDR adjusted p-value ≤ 0.1 and log2 fold change (FC) ≥ 0.5). Overall, 0.8% of the aphid genome was significantly impacted by the presence of PLRV.

**Table 1:** RNAseq stats.

Sample name	Total paired-end reads	Total alignments	Aligned	Unique paired	Non-unique paired
Control 33	40,676,571	30,990,077	76.17%	28.52%	47.67%
Control 34	18,942,570	13,440,120	70.80%	25.89%	45.07%
Control 35	22,651,445	16,478,122	72.49%	25.93%	46.82%
PLRV 36	25,343,473	18,306,992	72.12%	26.30%	45.94%
PLRV 37	23,743,718	17,496,940	73.63%	27.15%	46.54%
PLRV 38	23,533,704	17,410,337	73.78%	27.05%	46.93%

### Functional roles of differentially expressed genes

**Figure 2:**
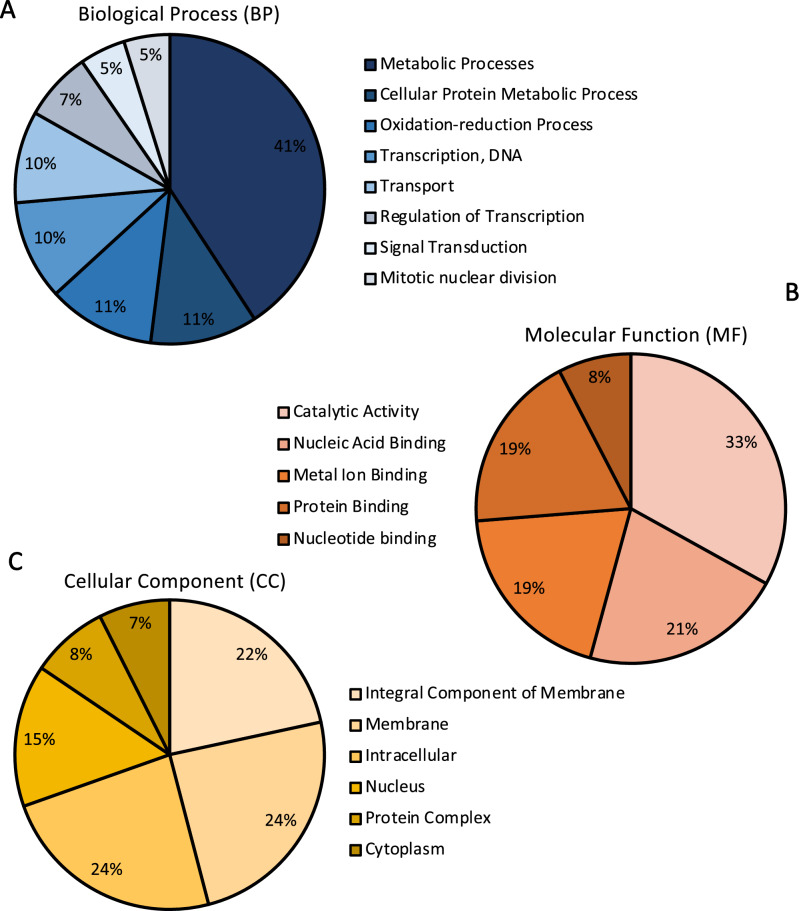
Blast2GO Gene Ontology of DEGs arranged by functional categories. **(A)** Biological processes (BP), **(B)** molecular function (MF), and **(C)** cellular component (CC). The predicted gene functions of differentially expressed genes as assigned by Blast2GO at level 2–3 in each aforementioned category. Each DEG may be assigned to one or more GOterm, with a total of 391 GOterms from the three functional groups assigned to the 134 DEGs.

Gene ontology (GO) enrichment analyses were performed with the DEGs from each treatment to identify functions and pathways disturbed in aphids carrying PLRV. One or more gene ontology terms were assigned to each transcript from biological processes, molecular functions, and cellular compartments term using Blast2GO functional gene annotation^49^. The 134 DEGs were assigned to functional GO terms within the three categories, including 125 biological processes, 118 molecular functions, and 148 cellular compartments. Of the 134 DEGs, 53 (39.55% of total DEGs) were classified as “uncharacterized proteins.” The majority of DEGs assigned to biological processes were categorized as metabolic processes (41%), cellular-protein processes (11%), and oxidation–reduction processes (11%) (Fig. 2A). As for DEGs assigned to molecular functions, almost half were associated with catalytic activity (33%) or nucleic acid binding (21%) (Fig. 2B). Within the cellular component category, 24% were related to the membrane and 24% were related to intracellular locations (Fig. 2C).

**Figure 3:**
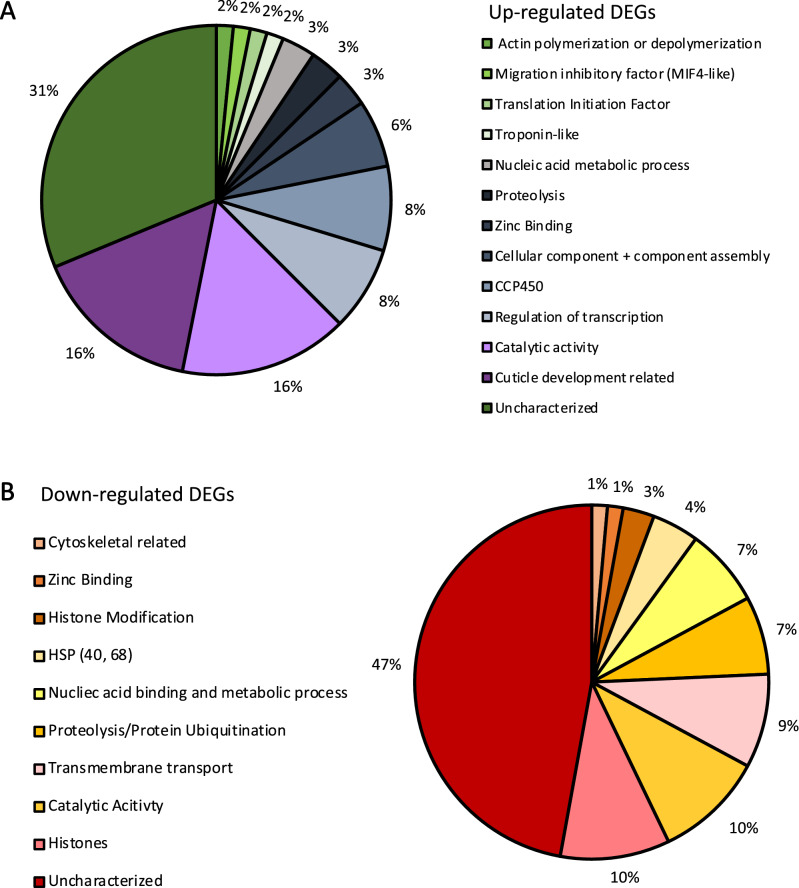
Blast2GO annotation for up-regulated DEGs and down-regulated DEGs in aphids carrying PLRV compared to controls. The consensus description predicted by Blast2GO of the **(A)** 64 up-regulated DEGs and the **(B)** 70 down-regulated DEGs.

Next each DEGs was annotated using a single Blast2GO consensus description. Many of the genes up-regulated in PLRV aphids were related to cuticle formation and development (16%), and catalytic activity (16%), however the majority of up-regulated transcripts were uncharacterized (31%; Fig. 3A). The largest groups of down-regulated genes in PLRV aphids were related to histones (10%), catalytic activity (10%), transmembrane transport (9%), proteolysis or protein ubiquitination (7%), and nucleic acid binding and metabolic processes (7%; Fig. 3B). A significant proportion of the down-regulated transcripts in PLRV aphids were also uncategorized (47%). The most highly expressed DEGs included transcripts related to cuticle formation and development, and 4C1-like cytochrome P450s (Table 2, Supplemental Table 2). The most down-regulated transcripts in PLRV aphids were related to histones and histone modifying proteins (Table 3).

**Table 2:** Most highly up-regulated *M. persicae* genes that were characterized [20 uncharacterized genes were up-regulated (see Supplementary Table S2)] in aphids carrying PLRV compared to controls.

Putative function	Gene ID	Blast2GO consensus description	p-val	log2
Cytochrome P450	000087490.2	cytochrome P450 4C1-like	1.30E−05	2.34
	000113270.1	cytochrome P450 4C1-like	1.90E−05	2.21
	000087490.3	cytochrome P450 4C1-like	3.10E−09	2.16
	000111320.1	cytochrome P450 6k1-like	1.10E−04	0.95
Cuticle related	**000133030.2**	**Adhesion plaque protein, chitin binding**	**4.60E**−**05**	**2.24**
	000133030.1	Adhesion plaque protein, chitin binding	1.90E−04	1.91
	000086070.1	Endocuticle glycoprotein in abdomen	3.00E−07	1.05
	000079280.1	Osiris 20-like	1.80E−06	0.95
	000103820.1	Adhesion plaque protein, chitin binding	6.60E−06	0.88
	000103820.2	Adhesion plaque protein, chitin binding	1.50E−06	0.87
	000079260.1	Osiris 18	9.40E−05	0.85
	000084640.1	Glycine and glutamine-rich	1.40E−05	0.77
	000047580.1	*Myzus persicae* tentative cuticle protein	2.60E−04	0.74
Kinase inhibitor repressors	000156640.1	52 kDa repressor of kinase inhibitor-like	7.10E−05	0.88
	000156640.2	52 kDa repressor of kinase inhibitor-like	3.70E−04	0.79
	000156640.4	52 kDa repressor of kinase inhibitor-like	3.50E−04	0.78
Kinases	000073070.1	Alpha-kinase 1-like	2.20E−06	0.78
	000137500.2	Serine threonine- kinase (NEK3)	2.20E−06	0.76
Hydrolase	000181580.1	*N*-acetylmuramoyl-l-alanine amidase-like	4.40E−04	0.93
Transcription factor	000125820.1	Transcription factor A2 (mab3-liked)	1.10E−05	1.94
Zinc transport	000174630.1	39S ribosomal mitochondrial	8.60E−05	0.77
Membrane	000137380.1	Histidine-rich glycoprotein	3.00E−05	0.90
Cell organization	000090710.1	Cytoskeleton-regulatory complex (pan1-like)	4.80E−04	1.268
	000021370.2	Microtubular process (CFA58-like)	4.70E−04	1.265
	000189110.1	Actin reorganization (WAS-like)	4.50E−04	0.83

DEGs determined by adjusted p value < 0.1 and described by Blast2GO. Gene ID corresponds to MYZPE13164_G006_v1.0_XXXXXXXXX.X found on AphidBase.org. Regulation of bolded transcripts were validated in a separate experiment.

**Table 3:** Most highly down-regulated *M. persicae* transcripts that were characterized [33 uncharacterized genes were down-regulated (see Supplemental Table S2)] in aphids carrying PLRV compared to controls.

Putative function	Gene	Blast2GO consensus description	p-value	log2
Histones	000100490.1	Histone H3	5.06E−05	−2.58
	000100610.1	Histone H3	2.69E−04	−2.14
	000100770.1	Histone H3	1.04E−04	−1.74
	000100600.1	Histone H2A	1.38E−04	−1.75
	000092680.1	Histone H2A-like	9.76E−05	−1.47
	000100590.1	Histone H2B-like	7.37E−05	−1.18
	000100620.1	Histone H4	3.02E−04	−0.96
Histone modifying	000163990.2	Glycine-rich DOT1-like	7.33E−05	−0.81
	000163990.1	Glycine-rich DOT1-like	1.97E−04	−0.76
Ubiquitination	000119640.3	E3-ubiqutin ligase RNF19B-like	6.08E−06	−0.88
	000119640.1	E3-ubiqutin ligase RNF19B-like	1.42E−05	−0.81
	000119640.2	E3-ubiqutin ligase RNF19B-like	2.34E−05	−0.78
Hydrolase	000133360.1	Serine carboxypeptidase	1.10E−04	−1.58
	000083200.2	Arylsulfatase B-like	4.69E−04	−1.11
	000083200.1	Arylsulfatase B-like	3.25E−04	−1.02
	000200070.1	Thioesterase (THEM6-like)	1.13E−04	−0.82
Response/immunity	000071560.1	Protease inhibitor (Papain inhibitor)	2.91E−04	−2.46
	**000070430.1**	**Heat shock 68-like**	**4.85E**−**04**	−**1.88**
	000193260.2	G-coupled receptor Mth-like 3	2.03E−04	−0.80
Transport	000029670.1	Dynein intermediate chain-like	1.90E−05	−1.01
	NRF6	Lipid transport (NRF6-like)	5.18E−05	−0.85
	000203490.1	Zinc finger C3H1 type-like 2-A	1.03E−04	−0.86
	000072950.2	Sugar transport (TRET1-like)	6.74E−06	−0.81
Nucleic acid metabolism	000036830.1	DNA integration (pol poly retrotransposon-related)	1.86E−04	−0.89
	000012610.1	mRNA catabolic process (BRISC/BRCA1-A complex-like)	5.14E−04	−0.84

DEGs determined by adjusted p value < 0.1 and described by Blast2GO. Gene ID corresponds to MYZPE13164_G006_v1.0_XXXXXXXXX.X found on AphidBase.org. Regulation of bolded transcripts were validated in a separate experiment.

### Validation of select aphid transcripts via RT-qPCR

**Figure 4:**
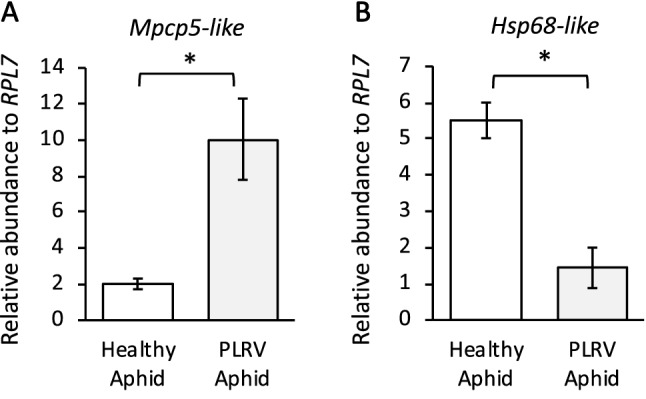
Relative transcript abundance of two genes in *Myzus persicae* with and without *Potato leafroll virus* (PLRV). **(A)** A cuticle related protein (*Mpcp5*-like) transcript was significantly up-regulated in expression in individuals with PLRV compared to virus free controls. **(B)** A predicted heat shock protein (*Hsp68*-like) was significantly down-regulated in expression in individuals with PLRV compared to controls. Transcripts were measured relative to a housekeeping gene *RPL7*. Significant differences were calculated using an ANOVA (*P < 0.05; Error bars represent ± SEM).

To validate the RNAseq we conducted qRT-PCR analysis of select DEGs in a separate experiment using the same experimental design. We selected one of the most up-regulated genes (MYZPE13164_G006_v1.0_ 000133030.2 (*Mpcp5-like*)) and one of the most down-regulated gene (MYZPE13164_G006_v1.0_ 000070430.1 (*Hsp68-like*)) and measured transcript abundance using qRT-PCR (bolded genes in Tables 2 and 3). *Mpcp5-like* is related to cuticle formation and development and *Hsp68-like* encodes a heat shock protein, which are related to immunity and stress responses. Consistent with the RNA-seq data, abundance of the *Mpcp5-like* transcript was significantly higher in viruliferous *M. persicae* compared to virus-free controls (10.045, 2.017, relative expression respectively; p = 0.019; Fig. 4A). Abundance of the *Hsp68-like* transcript was significantly lower in viruliferous *M. persicae* when compared to virus-free controls (1.44, 5.51, relative expression respectively; p < 0.01) (Fig. 4A,B).

### The impact of PLRV on *Buchnera aphidicola* titer

**Figure 5:**
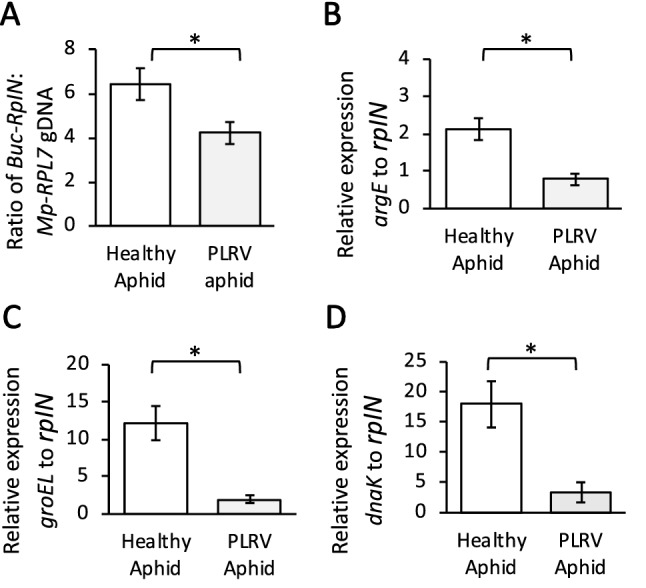
*Buchnera aphidicola* titer and transcript changes in *Myzus persicae* with *Potato leafroll virus* (PLRV). **(A)** Ratio of a single copy *Buchnera* gene *rpIN* relative to a single copy aphid gene *RPL7,* demonstrates *M. persicae* with PLRV have a decreased *Buchnera aphidicola* titer relative to control aphids. **(B–D)** *Buchnera* transcripts *groEL*, *dnaK*, and *argE* relative to the *Buchnera* gene *rpIN* housekeeping gene. All three transcripts were down-regulated in viruliferous *M. persicae* compared to virus free controls. Significant differences were calculated using an ANOVA (*P < 0.05; Error bars represent ± SEM).

*Buchnera* has been previously implicated in transmission of PLRV and other luteoviruses^23,25,27,31,54^, however *Buchnera* titer and coding sequence transcripts have not been examined in aphids carrying PLRV. From our experiments, *Buchnera* titer was ~ 1.5 times higher for virus-free aphids compared to aphids carrying PLRV (ratios 6.42, 4.20 respectively; p = 0.037; Fig. 5A). To investigate the potential mechanisms mediating decreases in *Buchnera* titer we measured abundance of two transcripts related to stress, *dnaK*^55,56^ and *groEL*^56,57^, and one transcript related to metabolism, *argE*^58^. Abundance of all three transcripts were reduced in aphids carrying PLRV compared to controls. Viruliferous aphids had 63.32% less *argE* transcripts (p = 0.026), 83.33% less *groEL* transcripts (p = 0.024), and 81.23% less *dnaK* transcripts (p = 0.046) compared to that of the virus free aphids (Fig. 5B–D).

## Discussion

The main focus of this paper was to examine the effect that *Potato leafroll virus* has on the transcriptome of *M. persicae,* and their primary endosymbiont *Buchnera aphidicola*. The largest category of known up-regulated transcripts in viruliferous aphids compared to controls were related to the cuticle and cuticle development. Insect cuticles are largely composed of a protein matrix embedded with chitin filaments^59^. Cuticle proteins (CPs) have been shown to be involved in general development, molting, transmission of non-persistent viruses, and insecticide resistance through changes in cuticle permeability^30,60–63^. In *Acyrthosiphon pisum*, 19 CPs were found to be regulated by photoperiodism and suspected to be involved in the transition from asexual to sexual production^64^. Further, cuticle proteins have been implicated as potentially facilitating transmission of *Barley yellow dwarf virus* (BYDV-GPV), *Cereal yellow dwarf virus* (CYDV-RPV), and *Turnip yellows virus* (TuYV), three related Luteoviridae viruses^65–67^. Whilst we cannot know the function of changes in CP transcripts in PLRV-aphid interactions from these experiments, these genes represent promising targets for further investigation.

In addition to many cuticle related proteins, five *cytochrome P450s* genes were significantly up-regulated in viruliferous aphids compared to controls. Cytochrome P450s play important roles in hormone and pheromone metabolism but are more famous for their roles in the metabolism of insecticides and host plant chemicals. Polyphagous insects, like *M. persicae,* encounter many different hosts and tend to have a higher number of P450s related to the metabolism of allelochemicals compared to more specialized aphids^68^. Previous work has shown that a *cytochrome 450 gene* (*CYP6CY3*) was found to increase nicotine tolerance and aphid host adaptation^69,70^. It has been previously hypothesized that up-regulation of p450s could help insect vectors tolerate less desirable hosts which could be beneficial to the virus^71^.

Transcripts encoding a heat shock protein (HSP68-like) was among the most down-regulated in viruliferous aphids compared to controls. HSP68 is a member of the HSP70 family, which are important chaperone proteins that are known to be up-regulated in response to stress. One study found that the *Hsp70* from *Bemisia tabaci* is up-regulated after acquisition of *Tomato yellow leaf curl virus* (TYLCV)^72^. They went on to show that HSP70 protein can directly interact with TYLCV using in vitro studies and that they co-localize together in insect midgut cells using in situ hybridization. The authors suggest HSP70 may play an inhibitory role in virus transmission, as transmission was increased when whiteflies were fed HSP70 antibodies. Given *Hsp68-like* transcripts were down-regulated in aphids carrying PLRV in our study, it would be interesting to investigate if this has any impact on PLRV transmission. Porras et al.^73^ demonstrated that BYDV-PAV, a strain that is only transmitted by *Rhopalosiphum padi* (bird-cherry oat aphid), up-regulated the abundance of three *Hsp70* transcripts in the aphid vector. The authors found BYDV infection increases plant surface temperature and aphid heat tolerance, suggesting a protective role of HSP70 proteins in virus-aphid-plant interactions^73^. Although it is not known if PLRV increases plant surface temperature and vector heat tolerance, it has been shown that potato plants kept at higher temperatures are more susceptible to PLRV than compared to lower temperatures^74^. Also aphid acquisition and transmission at higher temperatures resulted in higher transmission rates compared to lower temperatures^75^, however at very high temperatures differences were reduced^76^. It is not known how decreases in *Hsp68-like* transcripts in aphids carrying PLRV may alter aphid heat tolerance.

In this study there was a significant reduction of *Buchnera* titer and *Buchnera* gene expression of three genes (*dnaK*, *groEL,* and *argE*) in aphids carrying PLRV compared to control aphids. In general, gene regulation at the mRNA level in *Buchnera* is thought to be minimal because *Buchnera* transcription factors are reduced^58^ and very few transcriptional responses had been observed previously^77^. Only two transcription initiation factors ($\sigma$ 32 and $\sigma$ 70), the heat shock and housekeeping transcription factors, respectively, remain in *Buchnera* Myzus’s genomes^78^ similar to other *Buchnera* taxa^79,80^. The housekeeping sigma factor ($\sigma$ 70) initiates transcription of *argE* which is regulated by the repressor ArgR when bound to arginine in *Escherichia coli*^81^. Similar to other *Buchnera* taxa, *Buchnera* Myzus’s genome^78^ has lost the repressor ArgR so it is unclear how this gene is down-regulated in virus-infected aphids compared to un-infected aphids. The other two *Buchnera* genes (*dnaK* and *groEL*) that were down-regulated in this study in aphids carrying PLRV compared to control aphids are associated with the heat shock regulon^80^. Moreover, these *Buchnera* genes still retain recognizable $\sigma$ 32 promoter sites up-stream of *dnaK* and *groEL* in the Myzus *Buchnera* G006 genome (NCBI Reference Sequence: NZ_MJNC01000001; Supplemental Table 3) similar to other *Buchnera* taxa^80^. The $\sigma$ 32 heat shock response is highly conserved in bacteria and is initiated in response to stress, such as heat shock or other environmental stressors that destabilize proteins^78–81^. In this study it is unclear how PLRV is either directly or indirectly dampening *Buchnera*’s expression of *dnaK* and *groEL* and if it is through a similar mechanism that is also down-regulating the aphid’s stress response genes including *Hsp70*.

A decrease in *Buchnera* titer has previously been associated with different aphid clones^83^, plant diets^13^, increasing aphid nymphal age^82,84^, and heat shock^81,84,86^. Most obligate pathogens and symbionts, including *Buchnera,* overexpress the protein GroEL during non-heat shock conditions to rescue misfolded proteins^87^. We hypothesize that PLRV is reducing *Buchnera*’s ability to up-regulate genes that are associated with the heat shock regulon (Fig. 5C,D) and this may lead to increased stress, lysing of *Buchnera* cells, and ultimately a reduction of *Buchnera* titer (Fig. 5A). Other insect-plant pathogen systems are known to modulate obligate symbiont titer. For example, in whiteflies Portiera titer is modulated by the co‐occurrence of its facultative symbiont *Rickettsia* and TYLCV^88^. Alternatively, as PLRV-infected plants have higher concentrations of free amino acids^38^, the change in host plant diet may have influenced *Buchnera* titer similar to Zhang et al.^13^. Because aphids carrying PLRV may obtain higher levels of essential amino acids from virus-infected plants, *Buchnera* genes that are involved in arginine biosynthesis, such as *argE*, may be down-regulated compared to aphids feeding on un-infected plants with lower amounts of essential amino acids.

Parasites of plants and animals can modify host behavior to improve their own transmission and survival^6,7,89,90^. This work explores the complex relationships that exist between hosts, viruses, vectors, and endosymbionts, and opens up more questions regarding the complexity and depth of these relationships. Aphids and bacterial endosymbionts may benefit from relationships with plant-infecting viruses indirectly or directly but additional studies are needed. Although it is known that *Buchnera* titer and gene expression responses vary with aphid linages^91^, it is not known how this is impacted by long term associations with plant-infecting viruses. In regions where virus pressure is high or where poor hosts dominate, aphids may more often be associated with plant infecting viruses. This study also expands on previous work that given the mounting evidence of virus manipulation of insect vectors, this could have lasting impacts on the population structures of these insect vectors and their obligate endosymbiont.

## Supplementary Information

Supplementary Information.
