# Supplementary material for: Potato leafroll virus reduces Buchnera aphidocola titer and alters vector transcriptome responses
Source: Sci Rep. 2021 Dec 14;11:23931. doi: 10.1038/s41598-021-02673-6 (PMC8671517; doi:10.1038/s41598-021-02673-6)
Supplement: Supplementary file 1 — Supplementary Information. [file 41598_2021_2673_MOESM1_ESM.pdf]

Supplementary Table 1. Primers used in this study

Primer ID / Gene	Species	Sequence
arge F	*Buchnera*	GACACCATCATCAGGATTGCT
arge R	*Buchnera*	AGAAACGGATATGTCTGGAGCC
dnakf	*Buchnera*	AAACCGTAAGCAAGTGCAGC
dnakr	*Buchnera*	GCGGTTATTACAGTGCCTGC
groel F	*Buchnera*	AGGTGTAGTTGCTGGTGGTG
groel R	*Buchnera*	ACGTAACGGAGCTTCCATCG
MpBuc_rpIN_F	*Buchnera*	CGG GTA CCA ATA GGT TGC TC
MpBuc_rpIN_R	*Buchnera*	CGA TAT GCA AAC ATT GGT GAT GT
Mp_Rpl7 (cDNA)	*M. persicae*	TGC CGG AGT CTG TAC TCA AA
Mp_Rpl7 (cDNA)	*M. persicae*	CAG GCG TTC TTT ACG TTC CT
Mp_Rpl7 (gDNA)	*M. persicae*	CCGAGGCTTATGTTAAGGAA
Mp_Rpl7 (dDNA)	*M. persicae*	AATTTTGGTTCTGGGGGAAT
Mp_HSP68	*M. persicae*	GGACAAGGGAGACATAC
Mp_HSP68	*M. persicae*	TTGATGGACAGGTTGAG
Mp_Cuticle	*M. persicae*	GAAGATGACGGCCAATA
Mp_Cuticle	*M. persicae*	GCTGGGATGTAGGATTT
Eubact16S-23S-10F	*ribosomal RNA intergenic spacer*	AGTTTGATCATGGCTCAGATTG
Eubact16S-23S-480R	*ribosomal RNA intergenic spacer*	CACGGTACTGGTTCACTATCGGTC

Supplementary Table 2. List of significantly different transcripts between PLRV and control *Myzus persicae*.

	baseMean	log2FoldChange	lfcSE	stat	pvalue	padj
MYZPE13164_G006_v1.0_000086770.1	7.348144	-6.2691	1.491413	-4.20347	2.63E-05	0.013248
MYZPE13164_G006_v1.0_000094310.1	10.91588	-3.88003	1.028026	-3.77425	0.00016	0.045037
MYZPE13164_G006_v1.0_000031210.1	27.20759	-3.75737	0.684385	-5.49014	4.02E-08	6.20E-05
MYZPE13164_G006_v1.0_000190440.1	192.0129	-2.88567	0.67632	-4.26672	1.98E-05	0.011134
MYZPE13164_G006_v1.0_000166250.1	408.7837	-2.82953	0.263032	-10.7574	5.47E-27	1.35E-22
MYZPE13164_G006_v1.0_000100490.1	31.953	-2.58097	0.636822	-4.05289	5.06E-05	0.020149
MYZPE13164_G006_v1.0_000071560.1	43.07904	-2.4609	0.679266	-3.62288	0.000291	0.06746
MYZPE13164_G006_v1.0_000085230.1	14549.45	-2.30698	0.385321	-5.98717	2.14E-09	3.77E-06
MYZPE13164_G006_v1.0_000100610.1	51.38604	-2.13924	0.58715	-3.64344	0.000269	0.06388
MYZPE13164_G006_v1.0_000166240.2	13036.7	-2.01981	0.203812	-9.91013	3.76E-23	4.64E-19
MYZPE13164_G006_v1.0_000094050.1	624.1545	-1.945	0.266907	-7.28719	3.16E-13	9.77E-10
MYZPE13164_G006_v1.0_000126820.1	513.6829	-1.8861	0.505645	-3.73008	0.000191	0.049826
MYZPE13164_G006_v1.0_000070430.1	4624.369	-1.87777	0.538252	-3.48864	0.000485	0.092939
MYZPE13164_G006_v1.0_000137240.1	25634.06	-1.87091	0.422281	-4.43049	9.40E-06	0.007036

MYZPE13164_G006_v1.0_000166220.2	11655.83	-1.81747	0.209496	-8.67547	4.12E-18	2.59E-14
MYZPE13164_G006_v1.0_000073460.1	34.09633	-1.8141	0.486168	-3.73143	0.00019	0.049826
MYZPE13164_G006_v1.0_000166220.1	11868.3	-1.8062	0.211285	-8.54863	1.25E-17	6.15E-14
MYZPE13164_G006_v1.0_000166230.1	10136.92	-1.78765	0.218182	-8.19338	2.54E-16	1.05E-12
MYZPE13164_G006_v1.0_000166240.1	2493.127	-1.76884	0.235066	-7.52484	5.28E-14	1.86E-10
MYZPE13164_G006_v1.0_000100600.1	102.6103	-1.75484	0.460395	-3.81161	0.000138	0.040112
MYZPE13164_G006_v1.0_000100770.1	73.88868	-1.74128	0.44879	-3.87994	0.000104	0.033493
MYZPE13164_G006_v1.0_000085220.1	1723.653	-1.64148	0.189257	-8.67328	4.20E-18	2.59E-14
MYZPE13164_G006_v1.0_000190430.1	17959.16	-1.58145	0.229815	-6.8814	5.93E-12	1.33E-08
MYZPE13164_G006_v1.0_000133360.1	50.56754	-1.57553	0.407367	-3.8676	0.00011	0.033928
MYZPE13164_G006_v1.0_000137250.1	34478.21	-1.55845	0.38112	-4.08914	4.33E-05	0.018758
MYZPE13164_G006_v1.0_000092680.1	153.629	-1.47447	0.378416	-3.89643	9.76E-05	0.032578
MYZPE13164_G006_v1.0_000031200.1	10307.25	-1.39167	0.193314	-7.19901	6.07E-13	1.50E-09
MYZPE13164_G006_v1.0_000179180.1	268.7649	-1.32239	0.304734	-4.33949	1.43E-05	0.009043
MYZPE13164_G006_v1.0_000168140.1	129.1578	-1.30495	0.309973	-4.20988	2.56E-05	0.013146
MYZPE13164_G006_v1.0_000190420.1	9873.604	-1.21919	0.16934	-7.19964	6.04E-13	1.50E-09

MYZPE13164_G006_v1.0_000018650.1	390.5762	-1.21744	0.1946	-6.25611	3.95E-10	7.50E-07
MYZPE13164_G006_v1.0_000100590.1	207.7229	-1.17565	0.296571	-3.96414	7.37E-05	0.026363
MYZPE13164_G006_v1.0_000018650.2	121.9398	-1.13211	0.302552	-3.74189	0.000183	0.049826
MYZPE13164_G006_v1.0_000083200.2	217.9952	-1.11319	0.318231	-3.49804	0.000469	0.091443
MYZPE13164_G006_v1.0_000083200.1	287.5863	-1.02152	0.28417	-3.59477	0.000325	0.072891
MYZPE13164_G006_v1.0_000029670.1	266.669	-1.01331	0.23697	-4.27613	1.90E-05	0.01111
MYZPE13164_G006_v1.0_000100620.1	228.3113	-0.96491	0.267013	-3.61371	0.000302	0.068387
MYZPE13164_G006_v1.0_000101070.1	415.999	-0.91838	0.219211	-4.18947	2.80E-05	0.01381
MYZPE13164_G006_v1.0_000188970.1	1247.625	-0.90255	0.211255	-4.27231	1.93E-05	0.01111
MYZPE13164_G006_v1.0_000036830.1	1700.031	-0.8857	0.236995	-3.73719	0.000186	0.049826
MYZPE13164_G006_v1.0_000119640.3	1096.339	-0.87964	0.194462	-4.52345	6.08E-06	0.005181
MYZPE13164_G006_v1.0_000063620.1	1916.052	-0.87232	0.210548	-4.14309	3.43E-05	0.015966
MYZPE13164_G006_v1.0_000203490.1	6703.552	-0.85563	0.220367	-3.88276	0.000103	0.033493
MYZPE13164_G006_v1.0_000143330.1	1684.249	-0.84738	0.209369	-4.04728	5.18E-05	0.020311
MYZPE13164_G006_v1.0_000012610.1	208.3427	-0.84341	0.242832	-3.47321	0.000514	0.096419
MYZPE13164_G006_v1.0_000200070.1	492.0244	-0.8239	0.213435	-3.8602	0.000113	0.03412

MYZPE13164_G006_v1.0_000119640.1	1107.515	-0.81332	0.187398	-4.34007	1.42E-05	0.009043
MYZPE13164_G006_v1.0_000072950.2	646.0914	-0.81115	0.180185	-4.50178	6.74E-06	0.005368
MYZPE13164_G006_v1.0_000163990.2	6131.56	-0.80734	0.203594	-3.96543	7.33E-05	0.026363
MYZPE13164_G006_v1.0_000174420.1	33053.98	-0.80262	0.201117	-3.99081	6.58E-05	0.025154
MYZPE13164_G006_v1.0_000193260.2	289.9815	-0.79829	0.214861	-3.7154	0.000203	0.051124
MYZPE13164_G006_v1.0_000119640.2	1099.286	-0.77522	0.183282	-4.22964	2.34E-05	0.012845
MYZPE13164_G006_v1.0_000077140.1	462.2232	-0.76126	0.207869	-3.66219	0.00025	0.061142
MYZPE13164_G006_v1.0_000163990.1	5199.397	-0.75511	0.202861	-3.72231	0.000197	0.050257
MYZPE13164_G006_v1.0_000037450.2	733.9386	-0.75361	0.18892	-3.98906	6.63E-05	0.025154
MYZPE13164_G006_v1.0_000072950.1	671.1741	-0.74577	0.169469	-4.40062	1.08E-05	0.00768
MYZPE13164_G006_v1.0_000119640.4	1086.207	-0.74364	0.209887	-3.54305	0.000396	0.082777
MYZPE13164_G006_v1.0_000140350.1	491.4154	-0.73957	0.190452	-3.88324	0.000103	0.033493
MYZPE13164_G006_v1.0_000072950.3	601.1993	-0.70648	0.177245	-3.9859	6.72E-05	0.025154
MYZPE13164_G006_v1.0_000006940.1	569.4643	-0.70633	0.17415	-4.0559	4.99E-05	0.020149
MYZPE13164_G006_v1.0_000037450.1	743.0526	-0.63732	0.175079	-3.64018	0.000272	0.064076
MYZPE13164_G006_v1.0_000068700.3	646.8404	-0.6031	0.152954	-3.943	8.05E-05	0.028388
MYZPE13164_G006_v1.0_000053530.1	822.5586	-0.58183	0.154017	-3.77773	0.000158	0.044923

MYZPE13164_G006_v1.0_000046440.1	6495.17	-0.57534	0.150439	-3.82438	0.000131	0.038542
MYZPE13164_G006_v1.0_000055040.1	6593.961	-0.54492	0.133942	-4.06833	4.74E-05	0.01982
MYZPE13164_G006_v1.0_000183210.1	13036.32	-0.54233	0.126689	-4.28082	1.86E-05	0.01111
MYZPE13164_G006_v1.0_000068470.2	1768.426	-0.51045	0.146992	-3.47263	0.000515	0.096419
MYZPE13164_G006_v1.0_000199660.2	1279.934	-0.46762	0.135075	-3.46188	0.000536	0.098855
MYZPE13164_G006_v1.0_000199660.1	2261.918	-0.46553	0.131648	-3.53615	0.000406	0.083386
MYZPE13164_G006_v1.0_000065460.1	3689.208	-0.45395	0.125414	-3.61963	0.000295	0.06746
MYZPE13164_G006_v1.0_000098360.1	1320.296	0.488316	0.136544	3.576249	0.000349	0.076885
MYZPE13164_G006_v1.0_000029520.1	1225.54	0.502502	0.137161	3.663593	0.000249	0.061142
MYZPE13164_G006_v1.0_000113810.1	1731.694	0.502781	0.142119	3.53774	0.000404	0.083386
MYZPE13164_G006_v1.0_000160910.1	9169.591	0.50861	0.142549	3.567973	0.00036	0.077931
MYZPE13164_G006_v1.0_000001840.1	1709.087	0.511567	0.145273	3.521407	0.000429	0.08689
MYZPE13164_G006_v1.0_000101260.1	1541.262	0.514192	0.142001	3.621044	0.000293	0.06746
MYZPE13164_G006_v1.0_000109850.1	14769.5	0.52316	0.146387	3.57382	0.000352	0.076885
MYZPE13164_G006_v1.0_000113690.2	53420.24	0.531614	0.143657	3.700588	0.000215	0.053656
MYZPE13164_G006_v1.0_000122320.1	5110.663	0.533106	0.137986	3.863489	0.000112	0.034079
MYZPE13164_G006_v1.0_000175810.1	1816.076	0.547424	0.146123	3.746337	0.000179	0.049788
MYZPE13164_G006_v1.0_000081240.3	12126.49	0.54924	0.158127	3.473411	0.000514	0.096419
MYZPE13164_G006_v1.0_000109370.1	20003.89	0.563245	0.148012	3.805401	0.000142	0.040653
MYZPE13164_G006_v1.0_000078040.2	2853.942	0.571929	0.14106	4.054521	5.02E-05	0.020149

MYZPE13164_G006_v1.0_000078040.4	2808.5	0.581356	0.137821	4.218208	2.46E-05	0.012939
MYZPE13164_G006_v1.0_000042240.1	489.6125	0.590255	0.168692	3.498999	0.000467	0.091443
MYZPE13164_G006_v1.0_000179420.1	834.6325	0.614588	0.147748	4.159687	3.19E-05	0.015134
MYZPE13164_G006_v1.0_000138020.1	669.3269	0.623891	0.167409	3.726747	0.000194	0.049896
MYZPE13164_G006_v1.0_000198050.1	2393.871	0.626365	0.17166	3.648872	0.000263	0.063151
MYZPE13164_G006_v1.0_000057850.1	924.8512	0.641723	0.144808	4.431549	9.36E-06	0.007036
MYZPE13164_G006_v1.0_000143070.1	1215.805	0.645228	0.156094	4.133577	3.57E-05	0.016333
MYZPE13164_G006_v1.0_000078040.1	2828.397	0.652216	0.136145	4.790591	1.66E-06	0.001867
MYZPE13164_G006_v1.0_000078040.3	2864.032	0.666419	0.13235	5.035281	4.77E-07	0.000589
MYZPE13164_G006_v1.0_000007780.1	288.0747	0.68767	0.198428	3.465599	0.000529	0.098233
MYZPE13164_G006_v1.0_000002010.1	442.8698	0.688635	0.194172	3.546526	0.00039	0.08239
MYZPE13164_G006_v1.0_000188820.1	987.992	0.689925	0.179825	3.836658	0.000125	0.037108
MYZPE13164_G006_v1.0_000137500.1	1807.581	0.696201	0.179576	3.876916	0.000106	0.033493
MYZPE13164_G006_v1.0_000044350.1	3059.765	0.697995	0.178591	3.908349	9.29E-05	0.031797
MYZPE13164_G006_v1.0_000089540.1	730.4162	0.726994	0.152884	4.755192	1.98E-06	0.001958
MYZPE13164_G006_v1.0_000047580.1	9781.11	0.741331	0.20296	3.652601	0.00026	0.062851
MYZPE13164_G006_v1.0_000077410.1	564.1565	0.745981	0.181054	4.120202	3.79E-05	0.016996
MYZPE13164_G006_v1.0_000137500.2	1813.087	0.75589	0.15966	4.734371	2.20E-06	0.002038
MYZPE13164_G006_v1.0_000084640.1	21881.66	0.765192	0.176291	4.340512	1.42E-05	0.009043
MYZPE13164_G006_v1.0_000174630.1	262.2143	0.773421	0.196903	3.927938	8.57E-05	0.0298
MYZPE13164_G006_v1.0_000073070.1	9337.378	0.77744	0.164309	4.731558	2.23E-06	0.002038
MYZPE13164_G006_v1.0_000156640.4	534.3413	0.777969	0.217584	3.575496	0.00035	0.076885
MYZPE13164_G006_v1.0_000156640.2	334.8972	0.788342	0.221623	3.557127	0.000375	0.080028

MYZPE13164_G006_v1.0_000152800.1	493.4206	0.79073	0.212007	3.729742	0.000192	0.049826
MYZPE13164_G006_v1.0_000174380.1	1898.985	0.805479	0.226485	3.556438	0.000376	0.080028
MYZPE13164_G006_v1.0_000151150.1	1810.2	0.827213	0.181311	4.562397	5.06E-06	0.00446
MYZPE13164_G006_v1.0_000189110.1	342.7335	0.829529	0.2366	3.506047	0.000455	0.090578
MYZPE13164_G006_v1.0_000049150.1	10608.06	0.848591	0.13183	6.437026	1.22E-10	2.51E-07
MYZPE13164_G006_v1.0_000079260.1	3728.556	0.854414	0.218767	3.905592	9.40E-05	0.031797
MYZPE13164_G006_v1.0_000000580.1	13429.84	0.868046	0.205767	4.218591	2.46E-05	0.012939
MYZPE13164_G006_v1.0_000151150.3	1790.912	0.869982	0.18245	4.768337	1.86E-06	0.001911
MYZPE13164_G006_v1.0_000103820.2	38077.86	0.871511	0.181303	4.806926	1.53E-06	0.001802
MYZPE13164_G006_v1.0_000103820.1	11195.82	0.875892	0.194338	4.507044	6.57E-06	0.005368
MYZPE13164_G006_v1.0_000156640.1	327.5304	0.88303	0.222355	3.971269	7.15E-05	0.02635
MYZPE13164_G006_v1.0_000137380.1	1996.69	0.897918	0.21509	4.174625	2.98E-05	0.014453
MYZPE13164_G006_v1.0_000151150.4	1840.567	0.91197	0.175747	5.189119	2.11E-07	0.00029
MYZPE13164_G006_v1.0_000181580.1	143.101	0.925969	0.263584	3.51299	0.000443	0.088961
MYZPE13164_G006_v1.0_000151140.2	586.7578	0.9379	0.228187	4.11023	3.95E-05	0.01743
MYZPE13164_G006_v1.0_000111320.1	188.0281	0.953635	0.246327	3.871412	0.000108	0.033825
MYZPE13164_G006_v1.0_000079280.1	1906.186	0.954178	0.199632	4.779678	1.76E-06	0.001885
MYZPE13164_G006_v1.0_000151150.2	1790.441	0.993549	0.190802	5.207234	1.92E-07	0.000278
MYZPE13164_G006_v1.0_000086070.1	102929.4	1.050296	0.204963	5.124327	2.99E-07	0.000388
MYZPE13164_G006_v1.0_000021370.2	67.59859	1.265081	0.361747	3.497143	0.00047	0.091443
MYZPE13164_G006_v1.0_000090710.1	63.27992	1.267582	0.362799	3.493898	0.000476	0.091839
MYZPE13164_G006_v1.0_000113350.1	58.22296	1.291364	0.365361	3.534484	0.000409	0.083386
MYZPE13164_G006_v1.0_000133030.1	549.6315	1.910366	0.511931	3.731688	0.00019	0.049826

MYZPE13164_G006_v1.0_000125820.1	46.62938	1.935753	0.440062	4.398815	1.09E-05	0.00768
MYZPE13164_G006_v1.0_000087490.3	57.51336	2.164869	0.365392	5.92478	3.13E-09	5.15E-06
MYZPE13164_G006_v1.0_000113270.1	31.16485	2.213077	0.517668	4.275093	1.91E-05	0.01111
MYZPE13164_G006_v1.0_000133030.2	1346.195	2.242036	0.549871	4.077389	4.55E-05	0.019392
MYZPE13164_G006_v1.0_000087490.2	27.92179	2.339856	0.537216	4.355523	1.33E-05	0.009043

Supplementary Table 3. Heat shock promoter- Conserved σ32 -10 and -35 binding sites in *Escherichia coli* and *Buchnera* taxa*

Locus	Species	(-35)	(spacer)	(-10)
	Escherichia coli consensus	**CTTGAAA**	13-15 bp	**CCCCATNT**
*groLS***	*Buchnera*-Mp	**CTTGAAA**GTTTTGACAAAGAA**CCCTATAT**		
	Buchnera-Ap	**CTTGAAA**GTTTTAATAAATAT**CCCTATAT**		
	Buchnera-Sg	**CTTGAAA**GTTTTAGTAAGCAT**CCCTATAT**		
dnaKJ	*Buchnera*-Mp	**CTTGATG**ACCGGAAAAGTGA**CCCCATTT**		
	Buchnera-Ap	**CTTGATG**AGCGAAAAAGCGA**CCCCATTT**		
	Buchnera-Sg	**CTTGATG**ACGAGAAAAAAGA**CCCCATTT**		

* *Buchnera*-Sg data obtained from NCBI Reference Sequence: NZ_MJNC01000001; *E. coli*, *Buchnera*-Ap, Sg data obtained from Dunbar et al. 2007

** the *groEL groES* operon is also called *mopAB*

Referenc
e:

Dunbar H.E., Wilson A. C. C., Ferguson N. R., Moran N. A., 2007. Aphid thermal tolerance is governed by a point mutation in bacterial symbionts. PLoS Biol. 5: e96.

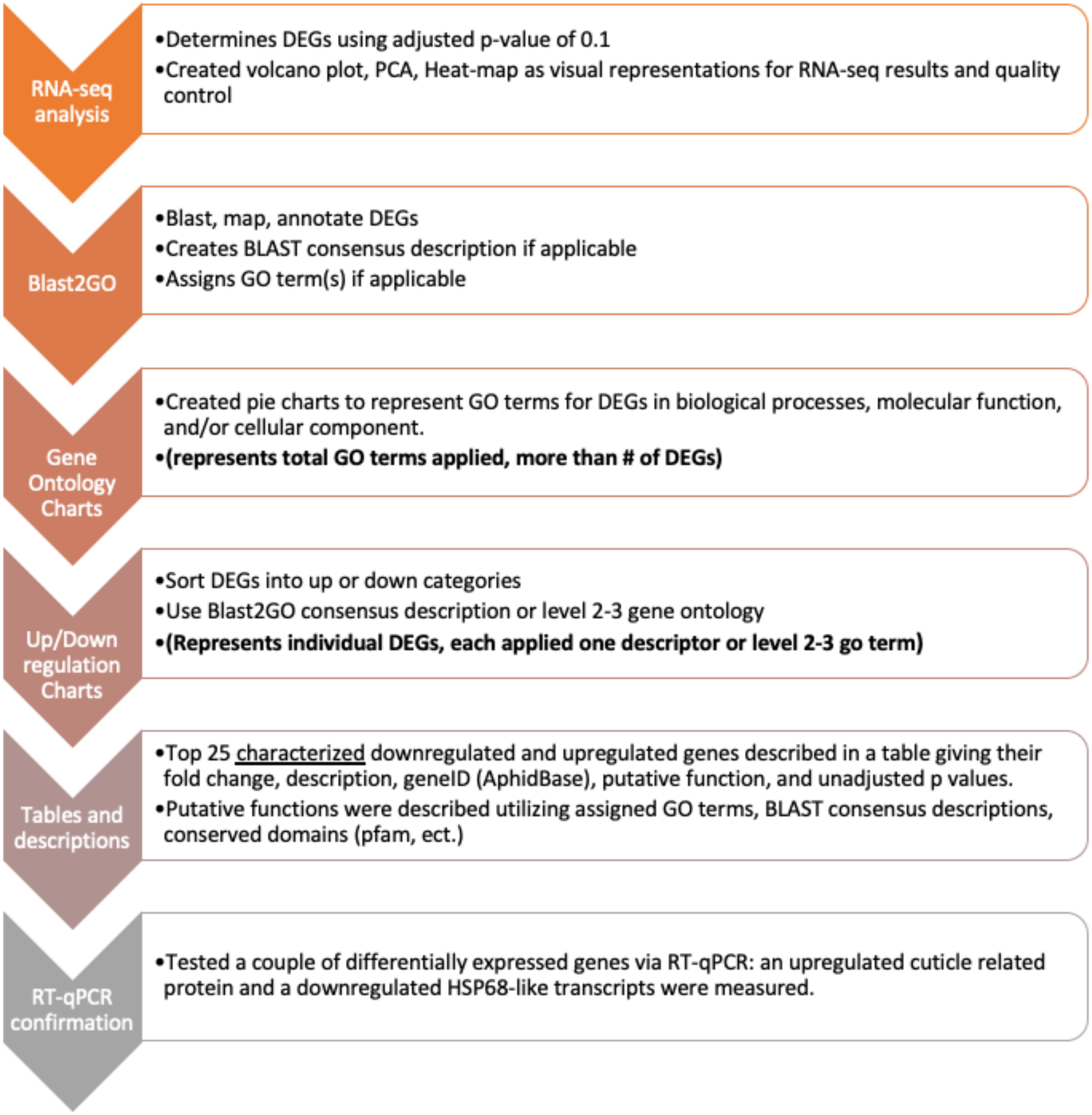

Figure S1: Explanation of process
